# Negatively charged residues in the first extracellular loop of the L-type Ca_V_1.2 channel anchor the interaction with the Ca_V_α2δ1 auxiliary subunit

**DOI:** 10.1074/jbc.M117.806893

**Published:** 2017-09-01

**Authors:** Benoîte Bourdin, Julie Briot, Marie-Philippe Tétreault, Rémy Sauvé, Lucie Parent

**Affiliations:** From the § Département de Pharmacologie et Physiologie, Faculté de Médecine, and; ‡ Centre de Recherche de l'Institut de Cardiologie de Montréal, Université de Montréal, Montréal, Québec H3C 3J7, Canada

**Keywords:** calcium channel, electrophysiology, gating, molecular modeling, protein-protein interaction

## Abstract

Voltage-gated L-type Ca_V_1.2 channels in cardiomyocytes exist as heteromeric complexes. Co-expression of Ca_V_α2δ1 with Ca_V_β/Ca_V_α1 proteins reconstitutes the functional properties of native L-type currents, but the interacting domains at the Ca_V_1.2/Ca_V_α2δ1 interface are unknown. Here, a homology-based model of Ca_V_1.2 identified protein interfaces between the extracellular domain of Ca_V_α2δ1 and the extracellular loops of the Ca_V_α1 protein in repeats I (IS1S2 and IS5S6), II (IIS5S6), and III (IIIS5S6). Insertion of a 9-residue hemagglutinin epitope in IS1S2, but not in IS5S6 or in IIS5S6, prevented the co-immunoprecipitation of Ca_V_1.2 with Ca_V_α2δ1. IS1S2 contains a cluster of three conserved negatively charged residues Glu-179, Asp-180, and Asp-181 that could contribute to non-bonded interactions with Ca_V_α2δ1. Substitutions of Ca_V_1.2 Asp-181 impaired the co-immunoprecipitation of Ca_V_β/Ca_V_1.2 with Ca_V_α2δ1 and the Ca_V_α2δ1-dependent shift in voltage-dependent activation gating. In contrast, single substitutions in Ca_V_1.2 in neighboring positions in the same loop (179, 180, and 182–184) did not significantly alter the functional up-regulation of Ca_V_1.2 whole-cell currents. However, a negatively charged residue at position 180 was necessary to convey the Ca_V_α2δ1-mediated shift in the activation gating. We also found a more modest contribution from the positively charged Arg-1119 in the extracellular pore region in repeat III of Ca_V_1.2. We conclude that Ca_V_1.2 Asp-181 anchors the physical interaction that facilitates the Ca_V_α2δ1-mediated functional modulation of Ca_V_1.2 currents. By stabilizing the first extracellular loop of Ca_V_1.2, Ca_V_α2δ1 may up-regulate currents by promoting conformations of the voltage sensor that are associated with the channel's open state.

## Introduction

In cardiac cells, Ca^2+^ signals control the force necessary for the myocardium to meet the physiological needs of the body (1). The primary pathway for Ca^2+^ influx in the adult heart is via the L-type Ca^2+^ channel. This Ca^2+^ influx pathway is essential for triggering sarcoplasmic reticulum Ca^2+^ release and is the major source of Ca^2+^ load in this organelle (2). Regulation of the L-type Ca^2+^ current has profound physiological significance. Alterations in density or the activation/inactivation gating of L-type Ca_V_1.2 channels have been implicated in hypertrophic signaling (3) and in a variety of cardiovascular diseases such as hypertension (4), atrial fibrillation (5–8), heart failure (9, 10), and congenital arrhythmias (11–13).

Cardiac L-type Ca_V_1.2 channels are heteromultimeric protein complexes formed by the pore-forming Ca_V_α1 subunit bound to the cytoplasmic Ca_V_β auxiliary subunit (14) with nanomolar affinity (15) and to the extracellular Ca_V_α2δ1 subunit (16–20). The Ca_V_α1 subunit is formed by a single polypeptide chain of 24 transmembrane helices grouped into four structural homologous repeats (repeats I, II, II, and IV). Ca_V_β promotes the cell-surface trafficking of Ca_V_1.2 channels through a single high-affinity binding site anchored on a tryptophan residue located in an intracellular helix linking repeats I and II of Ca_V_α1 (21).

**Figure 1.**
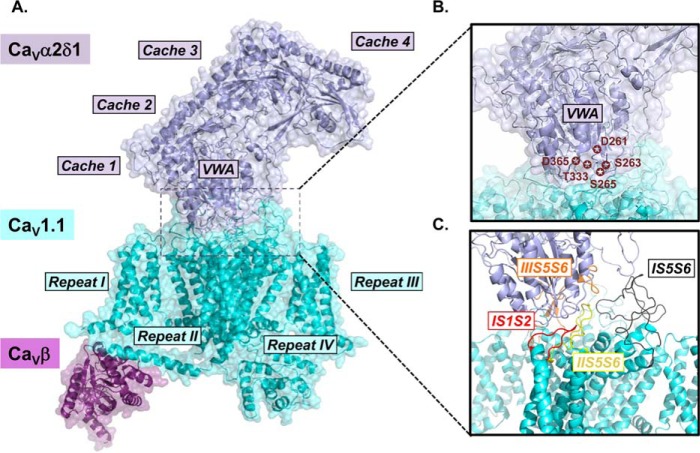
**Three-dimensional cryo-electron microscopy structure of the Ca_V_1.1 channel.** *A*, surface representation of the rabbit Ca_V_1.1 channel (*cyan*) in complex with Ca_V_α2δ1 (*deep blue*) and Ca_V_β2 (*violet*) (PDB: 5GJV). The structural domains of Ca_V_α2δ1 and the relative orientation of repeats I–IV in the pore-forming Ca_V_α1 subunit of Ca_V_1.1 are identified. For the correlation between the primary sequence and the structural domains of Ca_V_α2δ1, see Fig. 2 in Ref. 56. The extensive interface between the two proteins is surrounded by a *dashed black square,* and this region is shown enlarged in *B* and *C. B,* structural details of the VWA domain of Ca_V_α2δ1 are emphasized, and the residues forming the MIDAS are identified by *stars. C,* extracellular loops of Ca_V_1.1 forming the interface with Ca_V_α2δ1 are shown. Only the main chains are shown. The S1S2 loop in repeat I (IS1S2, residues 71–82) is in *red*; the turret and external pore region S5S6 in repeat I (IS5S6, residues 219–278) is in *black*; the turret and external pore region S5S6 in repeat II (IIS5S6, residues 581–600) is in *yellow*, and the turret and external pore region S5S6 in repeat III (IIIS5S6, residues 950–998) is in *orange*. Images were produced with PyMOL (Molecular Graphics System, Version 1.8 Schrödinger, LLC).

Co-expression of Ca_V_α2δ1 with Ca_V_β-bound Ca_V_α1 increases by up to 10-fold the peak current density and promotes the activation of Ca_V_1.2 at physiological voltages (22–27). The molecular determinants responsible for this modulation, and whether it involves a direct or an allosteric interaction, have yet to be identified. The three-dimensional (3D) structure of the homologous skeletal muscle Ca_V_1.1 channel from rabbit, solved by single particle cryo-electron microscopy (cryo-EM) (20, 28), proposes multiple protein interfaces between the two proteins (Fig. 1). Many clusters of residues within the extracellular von Willebrand factor A (VWA)^3^ and the cache1 domains of Ca_V_α2δ1 could form interactions with extracellular loops from repeats I–III of the Ca_V_α1 subunit of Ca_V_1.2. In particular, the polar residues Asp-261, Ser-263, Ser-265, Thr-333, and Asp-365 in the rabbit Ca_V_α2δ1 are forming the metal-ion-dependent adhesion site (MIDAS), which are purported to play important roles at the protein interface. These Ca_V_α2δ1 residues are facing residues in the extracellular loop linking the transmembrane helices S1 and S2 in the voltage sensor domain of repeat I (IS1S2) and the extracellular regions in the pore domains of repeats II and III (IIS5S6 and IIIS5S6, respectively), suggesting a broader interface than the highly localized α-interacting domain (AID) identified between Ca_V_1.2 and Ca_V_β (15, 21, 29).

In this work, we sought to identify the “hot spot AID” at the Ca_V_1.2/Ca_V_α2δ1 interface using a combination of protein chemistry, molecular modeling, and functional characterization of channel mutants. Structural alterations in the first extracellular loop in repeat I of Ca_V_1.2, by the insertion of the 9-residue human influenza hemagglutinin (HA = YPYDVPDYA) epitope, impaired the up-regulation of Ca_V_1.2 currents by Ca_V_α2δ1 and significantly decreased the co-immunoprecipitation of the two proteins. Single-point mutations of Asp-181 located within a conserved cluster of negatively charged residues (Glu-Asp-Asp or EDD) in the first extracellular loop of Ca_V_1.2 prevented the modulation of whole-cell currents and the co-immunoprecipitation of the two proteins. These data contrast with the modest impact of point mutations in the extracellular loop between the 5th and the 6th transmembrane helices in repeat III of Ca_V_1.2. Altogether, our data are compatible with a model where negatively charged aspartate residues in IS1S2 of Ca_V_1.2 anchor the interaction with Ca_V_α2δ1.

## Results

### Mapping the functional interface between Ca_V_1.2 and Ca_V_α2δ1

Functional regulation of Ca_V_1.2 by Ca_V_α2δ1 requires direct interaction between the two proteins (30). Surface mapping of the 3D structure obtained with the homologous skeletal muscle Ca_V_1.1 suggests that the two proteins share a complex interaction network (Fig. 1). Four hot spots are readily identifiable. Some residues in the extracellular loops IS1S2, IS5S6, and IIS5S6 of Ca_V_1.1 are located within atomic distance of residues in the VWA structural domain of Ca_V_α2δ1, whereas residues in IIIS5S6 of Ca_V_1.1 are closer to residues in the cache1 domain of Ca_V_α2δ1. From the close examination of the 3D structure, any or all of these interfaces could be involved in the functional modulation of Ca^2+^ currents. Functional modulation of the activation gating and increase in the peak current density could arise from the low- or moderate-affinity binding to require interaction with one or more of these interfaces such that Ca_V_α2δ1 could alternatively associate and dissociate from Ca_V_β-bound Ca_V_1.2 proteins (31). To determine whether any or all of these extracellular loops confer functional interaction, we evaluated the functional impact of inserting an HA tag from the human influenza virus, in extracellular domains of Ca_V_1.2. The HA tag was inserted after Ser-182 in IS1S2 (Ca_V_1.2–HA Ser-182), after Glu-331 in IS5S6 (Ca_V_1.2–HA Glu-331), and after Asp-710 in IIS5S6 (Ca_V_1.2–HA Asp-710). All these constructs were translated at the expected molecular mass and expressed at the cell surface as confirmed by a flow cytometry assay (results not shown) (25, 27, 32).

**Figure 2.**
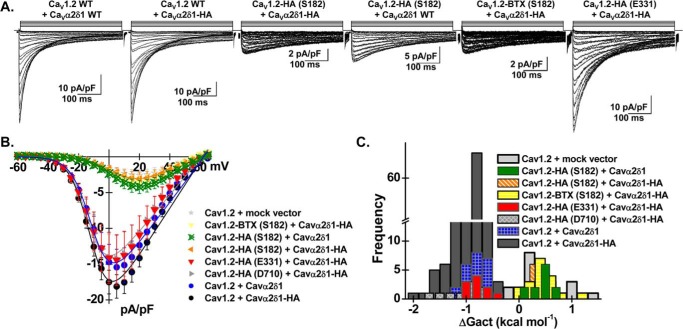
**Inserting epitopes within the first extracellular loop of Ca_V_1.2 prevents functional modulation of whole-cell current by Ca_V_α2δ1.** Stable recombinant HEKT cells expressing Ca_V_β3 were transiently transfected with pmCherry–Ca_V_α2δ1–HA (or pmCherry–Ca_V_α2δ1 WT) and pCMV–Ca_V_1.2 WT or mutants as indicated over each current trace. In all cases, Ca_V_α2δ1 was tagged in the C terminus by an mCherry epitope. The epitopes HA or BTX were inserted after the identified residues without altering the primary sequence on each side of the insertion site. *A,* representative whole-cell Ca^2+^ current traces obtained after recombinant expression of Ca_V_1.2 WT + Ca_V_α2δ1 WT, Ca_V_1.2 WT + Ca_V_α2δ1–HA, Ca_V_1.2-HA (Ser-182) + Ca_V_α2δ1–HA, Ca_V_1.2–HA (Ser-182) + Ca_V_α2δ1 WT, Ca_V_1.2–BTX (Ser-182) + Ca_V_α2δ1–HA, and Ca_V_1.2–HA (Glu-331) + Ca_V_α2δ1–HA (from *left to right*). Ca_V_1.2 currents were modulated to the same extent in the presence of mCherry–Ca_V_α2δ1–HA or mCherry–Ca_V_α2δ1. Furthermore, similar results were obtained with Ca_V_1.2–HA (Glu-331). Currents were recorded in the presence of 2 mm Ca^2+^ from a holding potential of −100 mV. Time scale is 100 ms throughout. The current density scale ranged from 2 to 10 pA/pF, as indicated. *B,* averaged current-voltage relationships. The absolute peak current densities measured with mCherry–Ca_V_α2δ1–HA varied from −4 to −46 pA/pF over the 12-month recording period with a mean of −18 ± 1 pA/pF (*n* = 243). Averaged peak current densities obtained with the mock mCherry vector are shown. Co-expression with Ca_V_α2δ1 left-shifted the voltage dependence of activation of Ca_V_1.2 WT/Ca_V_β3 from *E*_0.5, act_ = +8 ± 2 mV (*n* = 25) to *E*_0.5, act_ = −8.3 ± 0.2 mV (*n* = 243) for Ca_V_1.2 WT/Ca_V_β3 with mCherry–Ca_V_α2δ1–HA. Statistical analyses were performed with a one-way ANOVA test: *, *p* < 0.01, and **, *p* < 0.001, against the mock vector. See Table 1 for details. *C,* distribution of the free energies of activation. The free energies of activation (Δ*G*_act_) measured in the presence of the mock vector and in the presence of mCherry–Ca_V_α2δ1–HA are centered at 0.5 ± 0.1 and −0.78 ± 0.03 kcal mol^−1^, respectively. The distribution of the Δ*G*_act_ values for the following combinations Ca_V_1.2–HA (Ser-182) with Ca_V_α2δ1–HA, Ca_V_1.2-HA (Ser-182) with Ca_V_α2δ1 WT, and Ca_V_1.2–BTX (Ser-182) with Ca_V_α2δ1–HA overlapped with the Δ*G*_act_ values measured in the presence of the mock vector (no Ca_V_α2δ1).

**Figure 3.**
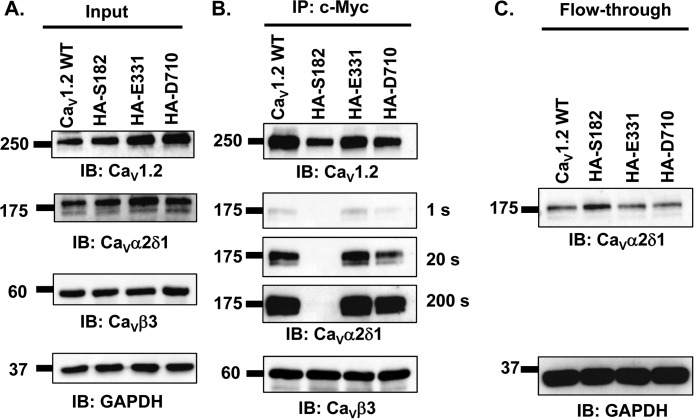
**Epitope insertion in the first extracellular loop of Ca_V_1.2 impairs the co-immunoprecipitation of Ca_V_α2δ1 with Ca_V_1.2/Ca_V_β3 proteins.** HEKT cells were transiently transfected with pmCherry–Ca_V_α2δ1–HA and pCMV–Ca_V_β3–c-Myc and either pCMV–Ca_V_1.2 WT, pCMV–Ca_V_1.2–HA (Ser-182), pCMV–Ca_V_1.2–HA (Glu-331), or pCMV–Ca_V_1.2–HA (Asp-710). Cell lysates were immunoprecipitated (*IP*) overnight with anti-c-Myc magnetic beads to capture Ca_V_β3, eluted in a Laemmli buffer, and fractionated by SDS-PAGE using 8% gels. *A,* immunoblotting was carried out on total proteins (20 μg) collected from the cell lysates for each of the three proteins (Ca_V_1.2, Ca_V_α2δ1, and Ca_V_β3) before the immunoprecipitation assay (*Input*) to confirm that each protein was translated at the expected molecular mass. Each experimental condition is identified by the specific Ca_V_1.2 construct. The signal for the housekeeping protein GAPDH is shown below each blot. *B,* immunoblotting (*IB*) was carried out after eluting the protein complexes from the anti-c-Myc beads with anti-Ca_V_1.2, anti-Ca_V_α2δ1, and anti-Ca_V_β3 antibodies (from *top to bottom*, as indicated). Images for Ca_V_α2δ1 were captured after short (1 s) or long exposure times (20 and 200 s). Ca_V_β3 and Ca_V_1.2 proteins migrated at 60 and 250 kDa, respectively. All Ca_V_α2δ1 proteins migrated at ≈175 kDa, which is consistent with the molecular mass of the mCherry–Ca_V_α2δ1–HA in previous studies (30). All immunoblots were carried out in parallel under the same transfection and extraction conditions. *C,* proteins that did not bind to the antibody-bead complex (referred to as the *flow-through* fraction) were collected, diluted in a Laemmli buffer, and fractionated by SDS-PAGE using an 8% gel and revealed with the anti-Ca_V_α2δ1. As seen, mCherry–Ca_V_α2δ1–HA is present in the flow-through fraction at the expected molecular mass (175 kDa) confirming that the proteins were appropriately translated and were present in the preparation in detectable quantities throughout. These experiments were carried out three times with the mutants and 10 times for the WT construct over a period of 5 months and yielded qualitatively similar results.

Whole-cell currents were recorded after recombinant expression of HA-tagged Ca_V_1.2 constructs with mCherry–Ca_V_α2δ1 wild-type (WT) or alternatively with mCherry–Ca_V_α2δ1–HA in stable Ca_V_β3 cells (Fig. 2*A*) (25, 27, 32). In all cases, co-expression of the complete set of subunits yielded high-voltage-activated Ca^2+^ currents. Co-expression of Ca_V_1.2 WT with the mCherry–Ca_V_α2δ1 WT construct enhanced whole-cell peak current densities by ≈7–10-fold from −2.5 ± 0.5 pA/pF (*n* = 25) (mock vector) to −15 ± 2 pA/pF (*n* = 23) in the presence of 2 mm Ca^2+^ (data not shown). Similar peak current densities of −18 ± 1 pA/pF (*n* = 243) were observed after co-expression with mCherry–Ca_V_α2δ1–HA (Fig. 2*B* and Table 1). The increase in peak current density was associated with a ≈ −20-mV leftward shift in the activation potential of Ca_V_1.2 (Fig. 2*C*) (25, 27). Co-expression of mCherry–Ca_V_α2δ1–HA with Ca_V_1.2–HA (Glu-331) or Ca_V_1.2–HA (Asp-710) yielded similar results with activation and inactivation kinetics not significantly different from that recorded for Ca_V_1.2 WT. The results obtained with Ca_V_1.2–HA (Asp-710) were reported before (25). In contrast, introduction of the HA epitope or the bungarotoxin (BTX) epitope “WRYYESSLEPYPD” (33) after Ser-182 impaired the stimulation of whole-cell Ca_V_1.2 currents by mCherry–Ca_V_α2δ1 WT and mCherry–Ca_V_α2δ1–HA as well as the hyperpolarizing shift in the activation potential. The patch-clamp data suggest that the modification of the primary sequence in the first extracellular loop IS1S2 in Ca_V_1.2 disrupts the interaction with Ca_V_α2δ1. To evaluate whether the insertion of the HA epitope disturbed the physical interaction between the two proteins, co-immunoprecipitation assays were carried out using c-Myc-tagged Ca_V_β3 on anti-c-Myc-coated beads as bait (Fig. 3) (30). These assays were carried out with the same quantity of total proteins after solubilization of full-length proteins with the non-ionic detergent digitonin, thus minimizing as much as possible the alterations in the complex network of interactions between the three proteins (20).

**Table 1:** **Biophysical properties of Ca_V_1.2 mutants** Ca_V_1.2 WT or mutant was co-expressed with Ca_V_β3 and either pmCherry–mock vector or pmCherry–Ca_V_α2δ1–HA WT or mutants using a 4:4:4 μg ratio. Biophysical parameters were measured in the presence of 2 mm Ca^2+^ as described elsewhere (25, 27). Activation properties (*E*_0.5, act_ and Δ*G*_act_) were estimated from the mean *I*–*V* relationships and fitted to a Boltzmann equation. Null-current cells outnumbered the cells with inward currents for Ca_V_1.2 D181R (12 null cells) and D181G (8 null cells). The data are shown as the mean ± S.E. of the number of cells (1 cell per experiment), and the total number of experiments carried over several months appears in parentheses. Statistical analysis was carried out against the values obtained in the presence of the mock pmCherry–N1 vector. NE means: no inward current;*, *p* < 0.01;**, *p* < 0.001.

Ca_V_1.2 WT or mutants with Ca_V_β3 and mCherry–Ca_V_α2δ1–HA	Electrophysiological properties in 2 mm Ca^2+^		
	Peak current density	*E*_0.5, act_	Δ*G*_act_
	*pA/pF*	*mV*	*kcal mol*^−*1*^
mCherry mock vector	−2.5 ± 0.3 (25)	+8 ± 2	+0.5 ± 0.1
Ca_V_1.2 WT	−18 ± 1 (243)**	−8.3 ± 0.2**	−0.78 ± 0.03**
Ca_V_1.2-BTX (Ser-182)	−2 ± 1 (18)	+6 ± 1	+0.4 ± 0.1
Ca_V_1.2-HA (Ser-182)	−5 ± 2 (10)	+7 ± 1	+0.5 ± 0.1
Ca_V_1.2-HA (Glu-331)	−17 ± 4 (10)**	−8.8 ± 0.5**	−0.8 ± 0.1**
Ca_V_1.2-HA (Asp-710)	−16 ± 2 (19)**	−8.8 ± 0.3**	−0.80 ± 0.04**
Ca_V_1.2 E179G	−27 ± 2 (8)**	−11 ± 1**	−1 ± 0.1**
Ca_V_1.2 E179A	−21 ± 3 (16)**	−5 ± 2**	−0.5 ± 0.1**
Ca_V_1.2 E179I	−20 ± 3 (9)**	−7 ± 1**	−0.6 ± 0.1**
Ca_V_1.2 E179R	−16 ± 3 (13)**	−6 ± 1**	−0.6 ± 0.1**
Ca_V_1.2 D180G	−18 ± 2 (18)**	−2 ± 1*	−0.04 ± 0.08*
Ca_V_1.2 D180I	−8 ± 1 (12)**	+3 ± 1	+0.24 ± 0.07
Ca_V_1.2 D180A	−9 ± 1 (14)**	+3 ± 1	+0.2 ± 0.1
Ca_V_1.2 D180E	−18 ± 3 (15)**	−3 ± 1*	−0.2 ± 0.1*
Ca_V_1.2 D181G	−3 ± 2 (3)	+7 ± 4	+0.4 ± 0.3
Ca_V_1.2 D181A	−4 ± 1 (8)**	+11 ± 6	+0.4 ± 0.2
Ca_V_1.2 D181R	−1 ± 1 (2)	+6 ± 1	+ 0.4 ± 0.1
Ca_V_1.2 D181E	−14 ± 3 (14)**	−4 ± 1*	−0.2 ± 0.1*
Ca_V_1.2 D180E/D181E	−24 ± 8 (8)**	+5 ± 2	+0.3 ± 0.2
Ca_V_1.2 S182G	−9 ± 2 (20)**	−7 ± 1**	−0.5 ± 0.1**
Ca_V_1.2 S182A	−23 ± 7 (9)**	−8 ± 2**	−0.8 ± 0.2**
Ca_V_1.2 S182R	−23 ± 7 (9)**	−7 ± 2**	−0.6 ± 0.1**
Ca_V_1.2 N183G	−11 ± 2 (9)**	−7 ± 2**	−0.7 ± 0.1**
Ca_V_1.2 N183A	−24 ± 6 (7)**	−11 ± 1**	−1 ± 0.1**
Ca_V_1.2 N183R	−13 ± 3 (7)**	−6 ± 1**	−0.5 ± 0.1**
Ca_V_1.2 N183Q	−7 ± 1 (7)**	−3 ± 2*	−0.3 ± 0.1*
Ca_V_1.2 A184G	−28 ± 7 (9)**	−11 ± 1**	−1.2 ± 0.2**
Ca_V_1.2 A184D	−22 ± 4 (9)**	−7 ± 2**	−0.8 ± 0.2**
Ca_V_1.2 A184R	−12 ± 3 (17)**	−7 ± 1**	−0.6 ± 0.1**
Ca_V_1.2 S957G	−26 ± 4 (6)**	−12 ± 1**	−1.3 ± 0.2**
Ca_V_1.2 K1100A	−19 ± 4 (9)**	−9 ± 1**	−0.9 ± 0.1**
Ca_V_1.2 K1100A + Ca_V_α2δ1–HA E174A	−14 ± 4 (9)**	−7 ± 1**	−0.6 ± 0.1**
Ca_V_1.2 I1104G	−21 ± 5 (10)**	−12 ± 2**	−1.1 ± 0.2**
Ca_V_1.2 G1109A	−14 ± 4 (10)**	−7 ± 1**	−0.7 ± 0.2**
Ca_V_1.2 H1113G	−26 ± 7 (8)**	−8 ± 2	−0.8 ± 0.2
Ca_V_1.2 R1119A	−12 ± 2 (31)**	−10 ± 1**	−0.9 ± 0.1**
Ca_V_1.2 R1119A + Ca_V_α2δ1–HA D171A	−10 ± 2 (9)**	−8 ± 1**	−0.7 ± 0.1**

The three proteins (Ca_V_1.2, Ca_V_α2δ1, and Ca_V_β3) were detected at the expected molecular mass in the total cell lysates (“input lanes”) when either Ca_V_1.2 WT, Ca_V_1.2–HA (Ser-182), Ca_V_1.2–HA (Glu-331), or Ca_V_1.2–HA (Asp-710) was co-expressed with Ca_V_β3 and mCherry–Ca_V_α2δ1–HA (Fig. 3*A*). Disruptions of the extracellular loops in Ca_V_1.2 did not impair the strong interaction with Ca_V_β3, and immunoblotting of the proteins bound to the beads revealed robust signals for Ca_V_1.2 even for Ca_V_1.2–HA (Ser-182) (Fig. 3*B*). However, the signal for Ca_V_α2δ1 that remained hooked onto the heteromeric complex was significantly weaker in the lysates prepared with Ca_V_1.2–HA (Ser-182) than with any other Ca_V_1.2 construct even after longer exposure times or after doubling the quantity of proteins loaded onto the anti-c-Myc-coated beads (data not shown). Ca_V_1.2–HA (Ser-182) was nonetheless clearly present in the flow-through (Fig. 3*C*) fraction. Altogether, these data indicate that disrupting the first extracellular loop IS1S2 in Ca_V_1.2 prevents the functional interaction with Ca_V_α2δ1 and suggest that the IS1S2 extracellular loop contains the elements that are important for carrying the physical interaction between the two proteins.

### Cluster of negatively charged residues is conserved in IS1S2 of Ca_V_1.2

**Figure 4.**
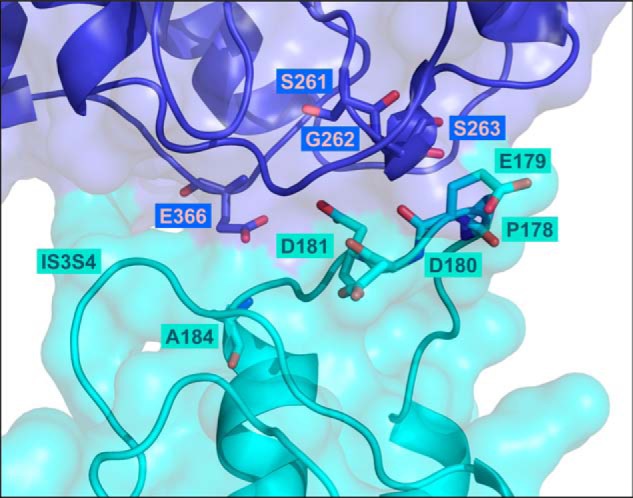
**Three-dimensional model of the extracellular loops of the rabbit Ca_V_1.2 in complex with the VWA domain of the rat Ca_V_α2δ1 protein.** The 3D model of the region spanning the first transmembrane helix S1 to the fourth transmembrane helix S4 in the first repeat in Ca_V_1.2 (IS1S4) is shown in *cyan,* and the VWA domain of Ca_V_α2δ1 is shown in *deep blue*. Residues Pro-178, Glu-179, Asp-180, Asp-181, and Ala-184 of Ca_V_1.2 and residues Ser-261, Gly-262, Ser-263, and Glu-366 in Ca_V_α2δ1 (three of the five residues in the MIDAS) are shown in *stick* representation with oxygen and nitrogen atoms colored in *red* and in *blue*, respectively. The model does not predict strong electrostatic interactions between Ca_V_1.2 Asp-180 and residues in Ca_V_α2δ1. Intramolecular interactions with residues in the extracellular IS3S4 loop are not ruled out. Ca_V_1.2 Asp-181 appears to be appropriately oriented to form electrostatic interactions with Gly-262 and Ser-263 in Ca_V_α2δ1. Modeling was achieved with Modeler 9.17. The figure was produced using PyMOL.

The insertion of a 9-residue epitope after Ser-182 in the 17-residue IS1S2 extracellular loop prevented the optimal interaction with Ca_V_α2δ1 by directly modifying the interaction site or indirectly from a nonspecific alteration of the secondary structure that was propagated to the actual interaction site. To narrow down the residues from Ca_V_1.2 that are potentially interacting with Ca_V_α2δ1, we run the bioinformatics tool PDBePISA (34) (http://www.ebi.ac.uk/pdbe/pisa/)^4^ using the molecular coordinates 5GJV.PDB of the whole-Ca_V_1.1 channel complex (69% residues are conserved with Ca_V_1.1 being shorter in the N and the C termini). This led to the identification of Glu-76, Asp-78, and Ser-81 in Ca_V_1.1 as likely candidates to form salt bridges with residues in the VWA domain of Ca_V_α2δ1. This first level of analysis suggests that the equivalent residues in the IS1S2 loop of Ca_V_1.2 could interact with Gly-262 and Ser-263 in Ca_V_α2δ1. To refine the structural predictions, a 3D model of the 146-residue region spanning from IS1 to IS4 in the rabbit Ca_V_1.2 was built using the molecular coordinates of Ca_V_1.1 (Fig. 4). A stretch of four residues ^74^PEDD^77^ in IS1S2 of Ca_V_1.1 is strictly conserved and is equivalent to ^178^PEDD^181^ in Ca_V_1.2. Ser-81 in Ca_V_1.1 corresponds to Ala-184 in Ca_V_1.2. The modeled interface suggests the region ^179^EDD^181^ in IS1S2 of Ca_V_1.2 lies within atomic distance (<5 Å) of many residues in the rat Ca_V_α2δ1. In particular, Ca_V_1.2 Asp-181 arises as the most likely partner for residues Gly-262 and Ser-263 in Ca_V_α2δ1.

### Ca_V_1.2 Asp-181 in IS1S2 anchors the physical interaction with Ca_V_α2δ1

**Figure 5.**
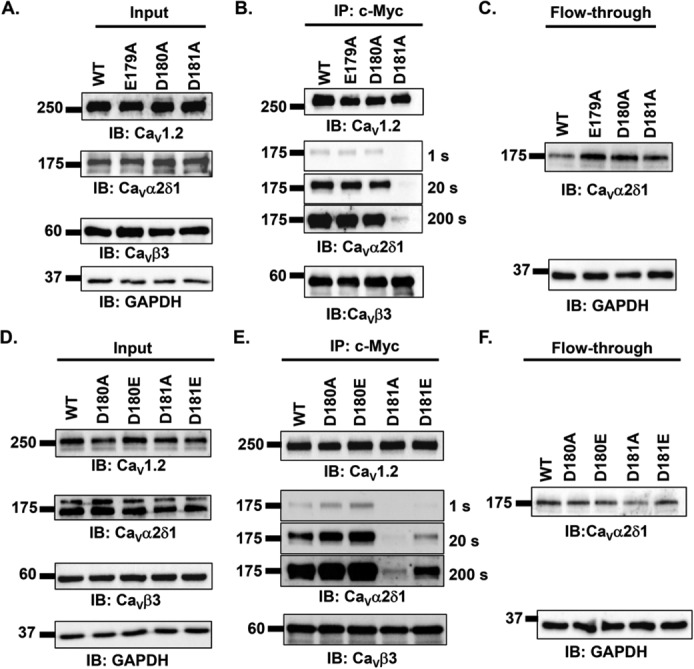
**Mutations of the aspartate residue at position 181 impair the co-immunoprecipitation of Ca_V_α2δ1 with Ca_V_1.2/Ca_V_β3 proteins.** *A–C,* HEKT cells were transiently transfected with pmCherry–Ca_V_α2δ1–HA and pCMV–Ca_V_β3–c-Myc and either pCMV–Ca_V_1.2 WT, pCMV–Ca_V_1.2 E179A, pCMV–Ca_V_1.2 D180A, or pCMV–Ca_V_1.2 D181A. Cell lysates were immunoprecipitated (*IP*) overnight with anti-c-Myc magnetic beads to capture Ca_V_β3, eluted in a Laemmli buffer, and fractionated by SDS-PAGE using 8% gels. *A,* immunoblotting was carried out on total proteins (20 μg) collected from the cell lysates for each of the three proteins (Ca_V_1.2, Ca_V_α2δ1, and Ca_V_β3) before the immunoprecipitation assay (*Input*) to confirm that each protein was translated at the expected molecular mass. Each experimental condition is identified by the specific Ca_V_1.2 construct. The signal for the housekeeping protein GAPDH is shown *below* each blot. *B,* immunoblotting was carried out after eluting the protein complexes from the beads with anti-Ca_V_1.2, anti-Ca_V_α2δ1, and anti-Ca_V_β3 antibodies (from *top to bottom*, as indicated). Images for Ca_V_α2δ1 were captured after short (1 s) or longer exposure times (20 and 200 s). All immunoblots were carried out in parallel under the same transfection and extraction conditions. *C,* proteins that did not bind to the antibody-bead complex (*flow-through fraction*) were collected, diluted in a Laemmli buffer, and fractionated by SDS-PAGE using an 8% gel and revealed with the anti-Ca_V_α2δ1. *D–F,* HEKT cells were transiently transfected with pmCherry–Ca_V_α2δ1–HA and pCMV–Ca_V_β3–c-Myc and either pCMV–Ca_V_1.2 WT, pCMV–Ca_V_1.2 D180A, pCMV–Ca_V_1.2 D180E, pCMV–Ca_V_1.2 D181A, or pCMV–Ca_V_1.2 D181E. Cell lysates were immunoprecipitated overnight with anti-c-Myc magnetic beads to capture Ca_V_β3, eluted in a Laemmli buffer, and fractionated by SDS-PAGE using 8% gels. *D,* immunoblotting was carried out on total proteins (20 μg) collected from the cell lysates for each of the three proteins (Ca_V_1.2, Ca_V_α2δ1, and Ca_V_β3) before the immunoprecipitation assay (*Input*) to confirm that each protein was translated at the expected molecular mass. Each experimental condition is identified by the specific Ca_V_1.2 construct. The signal for the housekeeping protein GAPDH is shown *below* each blot. *E,* immunoblotting (*IB*) was carried out with anti-Ca_V_1.2, anti-Ca_V_α2δ1, and anti-Ca_V_β3 antibodies (from *top to bottom*, as indicated) after eluting the protein complexes from the beads. Images for Ca_V_α2δ1 were captured after short (1 s) or longer exposure times (20 and 200 s). All immunoblots were carried out in parallel under the same transfection and extraction conditions. *F,* proteins that did not bind to the antibody-bead complex (*flow-through fraction*) were collected, diluted in a Laemmli buffer, and fractionated by SDS-PAGE using an 8% gel and revealed with the anti-Ca_V_α2δ1. As seen, mCherry–Ca_V_α2δ1–HA is present in the flow-through fraction at the expected molecular mass (175 kDa) confirming that the proteins were appropriately translated and were present in the preparation in detectable quantities throughout. These experiments were carried out four times with the mutants and 10 times for the WT construct over a period of 5 months and yielded reproducible results.

Because any of the three negatively charged residues could potentially interact with Ca_V_α2δ1, co-immunoprecipitation assays were carried out with single Ca_V_1.2 mutants E179A, D180A, and D181A to identify the role of each amino acid (Fig. 5, *A–C*). Again, all three proteins were detected at the expected molecular mass in the input lanes prepared from the total cell lysates. Immunoblotting of the proteins eluted from the c-Myc-coated beads revealed strong signals for Ca_V_1.2 under all conditions even for Ca_V_1.2 D181A. The signal for Ca_V_α2δ1 was easily detected in the presence of Ca_V_1.2 WT, Ca_V_1.2 E179A, and Ca_V_1.2 D180A. However, Ca_V_α2δ1 could only be detected after a 200-s exposure when co-expressed with Ca_V_1.2 D181A. These results were repeated even after doubling the quantity of Ca_V_1.2 D181A protein as the starting material (results not shown). The protein interaction appeared thus to be considerably weakened by the mutation in Ca_V_1.2. The properties of the side chain at position Asp-181 in Ca_V_1.2 were further explored with a conservative substitution with a negatively charged glutamate (109 Å^3^) (35). The latter is expected to cause minimal changes in steric hindrance with the addition of a single –CH_2_ group (Δ volume ≈17–18 Å^3^). As seen, all three proteins were detected at the expected molecular mass in the input lanes prepared from the total cell lysates under experimental conditions where Ca_V_1.2 WT, Ca_V_1.2 D180A, Ca_V_1.2 D180E, Ca_V_1.2 D181A, and Ca_V_1.2 D181E were co-expressed with Ca_V_β3 and mCherry–Ca_V_α2δ1–HA (Fig. 5*D*). Immunoblotting of the proteins bound to the anti-c-Myc-coated beads revealed strong signals for the anti-Ca_V_1.2 under all conditions indicating that all Ca_V_1.2 proteins (WT and mutants) were hooked onto the Ca_V_β3 beads. Ca_V_α2δ1 was found to hang onto Ca_V_1.2 WT, Ca_V_1.2 D180A, and Ca_V_1.2 D181E proteins as illustrated with bands of similar intensity detected after 1-s exposure (Fig. 5*E*). Again, Ca_V_α2δ1 did not bind to the c-Myc–Ca_V_β3–Ca_V_1.2 D181A complex with the same intensity and could only be detected after a 200-s exposure. Note that the interaction between the two proteins was slightly decreased with Ca_V_1.2 D181E despite the conservation of the negative charge, and the Ca_V_α2δ1 signal could only be revealed after a 20-s exposure. The unbound fraction was collected, and immunoblotting confirmed that Ca_V_α2δ1 was not in rate-limiting quantity. These results confirm that a negatively charged residue at position 181 in Ca_V_1.2 is required to promote the interaction with Ca_V_α2δ1 with a stronger affinity for the aspartate than the glutamate residue.

### Ca_V_α2δ1-mediated up-regulation of whole-cell currents is prevented by mutations of Ca_V_1.2 Asp-181

**Figure 6.**
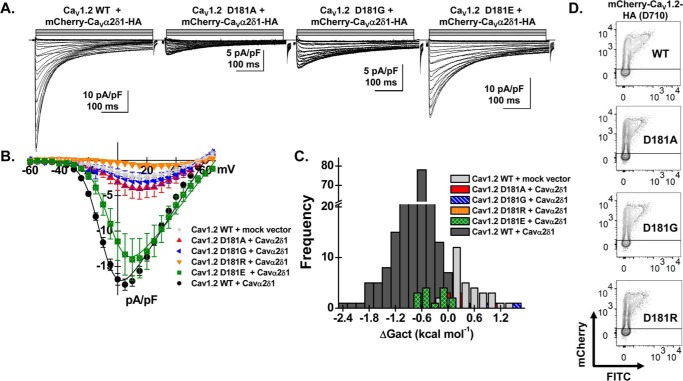
**Mutations at Asp-181 prevent up-regulation of Ca_V_1.2 currents.** HEKT cells were transiently transfected with pCMV–Ca_V_β3 and pmCherry–Ca_V_α2δ1–HA with the Ca_V_1.2 constructs (D181A, D181G, D181R, and D181E). *A,* whole-cell Ca^2+^ current traces were recorded in the presence of 2 mm Ca^2+^ from a holding potential of −100 mV for the constructs as identified. The current traces with the largest currents are shown for Ca_V_1.2 constructs D181G and D181E. Time scale is 100 ms throughout. The current density scale is either 5 or 10 pA/pF as indicated. *B,* averaged current-voltage relationships. Peak current densities *versus* voltage relationships were measured for Ca_V_1.2 WT and Ca_V_1.2 mutants (as shown). Currents traces obtained with the empty mCherry (*mock*) vector are also shown. Ca_V_1.2 constructs D181A, D181G, and D181R generated currents that were not significantly up-regulated by mCherry–Ca_V_α2δ1–HA WT. Statistical analyses were performed with a one-way ANOVA test: *, *p* < 0.01, and **, *p* < 0.001 against the mock vector. See Table 1 for details. *C,* distribution of the free energies of activation. The values for the free energy of activation (Δ*G*_act_) measured for Ca_V_1.2 constructs (D181A, D181G, D181E, and D181R) overlapped with the values measured for the mock vector. *D,* representative two-dimensional plots of mCherry *versus* FITC fluorescence. The cell-surface expression of the Ca_V_1.2 mutants was evaluated by introducing the mutation in the mCherry–Ca_V_1.2–HA construct. The surface fluorescence was estimated from the relative intensity of the fluorescence emitted by the fluorescein isothiocyanate (FITC)-conjugated anti-HA as measured using a flow cytometry assay (10,000 intact cells). The construct allows for detection of intracellular and extracellular fluorescence using FITC-conjugated anti-HA (“*x* axis”) and an anti-mCherry (“*y* axis”), respectively. The robust mCherry signal (*y* axis) confirms that the proteins were translated up to the end of the coding sequence. The cell-surface fluorescence for FITC, calculated as ΔMedFI as explained under “Experimental procedures,” was slightly lower for the Ca_V_1.2 mutants (D181A, D181G, and D181R) than for the WT construct. Nonetheless, all constructs significantly fluoresced at the cell surface supporting the view that the absence of function did not result from a complete absence of trafficking to the cell membrane. Furthermore, the ΔMedFI signal for the total protein was similar for all WT and mutant constructs demonstrating that proteins were appropriately translated, an observation also obtained from carrying out routine Western blotting.

The functional importance of the negatively charged side chain in conferring this interaction was confirmed in patch-clamp experiments performed with Ca_V_1.2 mutants. Substitutions were carried out at position 181 with glycine (48 Å^3^), hydrophobic alanine (67 Å^3^), and arginine (148 Å^3^) residues (35). In the presence of Ca_V_α2δ1, Asp-181 mutants (D181G, D181A, and D181R) produced voltage-activated inward Ca^2+^ current currents that were indistinguishable from currents produced with Ca_V_1.2 WT in the absence of Ca_V_α2δ1 (Fig. 6, *A–C*, and Table 1). The near-absence of voltage-activated currents with these mutants was observed despite their cell-surface expression (Fig. 6*D*). In contrast, whole-cell currents produced by Ca_V_1.2 D181E were up-regulated by Ca_V_α2δ1 indicating that the functional modulation requires a negative side chain at this position. Nonetheless, the activation of Ca_V_1.2 D181E was slightly right-shifted when compared with Ca_V_1.2 WT suggesting that up-regulation of whole-cell currents can be achieved without a significant modification of the activation gating.

### Ca_V_1.2 Asp-180 influences activation gating without changing peak current density

**Figure 7.**
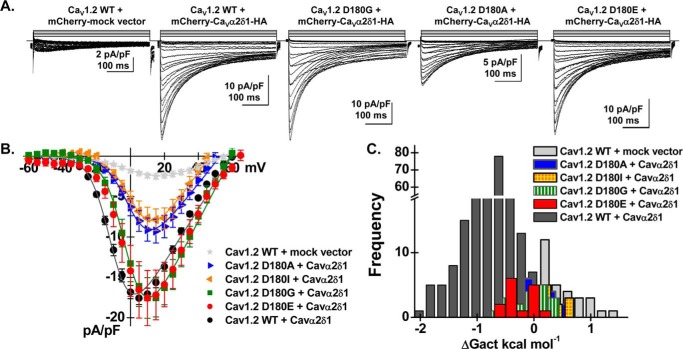
**Asp-180 in Ca_V_1.2 controls the Ca_V_α2δ1-induced shift in voltage-dependent gating of Ca_V_1.2 currents.** *A,* representative whole-cell Ca^2+^ current traces obtained after the transient expression of pCMV–Ca_V_β3 and pmCherry–Ca_V_α2δ1–HA with the Ca_V_1.2 constructs (D180I, D180A, D180G, and D180E) in HEKT cells. Note that the *leftmost* current trace was obtained in the presence of Ca_V_1.2 WT and the mock mCherry vector. Recordings were made in the presence of 2 mm Ca^2+^ from a holding potential of −100 mV. Time scale is 100 ms throughout. The current density scale ranged from 2 to 10 pA/pF as indicated. *B,* averaged current-voltage relationships. Peak current densities *versus* voltage relationships were measured for Ca_V_1.2 WT and Ca_V_1.2 mutants (as shown). Averaged peak current densities obtained with the mock mCherry vector are shown in *light gray stars*. All Ca_V_1.2 constructs (D180I, D180A, D180G, and D180E) were up-regulated by mCherry–Ca_V_α2δ–HA albeit to variable extent. Statistical analyses were performed with a one-way ANOVA test: *, *p* < 0.01, and **, *p* < 0.001, against the mock mCherry vector. Nonetheless, the current-voltage relationships measured with the Ca_V_1.2 mutants were clearly shifted to the right when compared with Ca_V_1.2 WT. See Table 1 for details. *C,* distribution of the free energies of activation. The free energies of activation (Δ*G*_act_) for Ca_V_1.2 D180E, D180I, D180A, and D180G did not overlap with the values measured with Ca_V_1.2 WT. Δ*G*_act_ values for Ca_V_1.2 D180A and D180I were not significantly different from Δ*G*_act_ values measured for the mock vector, whereas Δ*G*_act_ values for Ca_V_1.2 D180G and D180E were significantly different at *p* < 0.01.

In contrast to most mutations at position 181, Ca_V_1.2 Asp-180 mutants produced large high-voltage-activated inward Ca^2+^ currents in the presence of Ca_V_α2δ1 (Fig. 7 and Table 1). The stimulation of peak current densities by Ca_V_α2δ1 ranged from 3- to 7-fold with D180A ≈ D180I < D180G ≈ D180E ≈ WT. Mutations at these positions, except for D180E, activated in a range of voltages that overlapped with Ca_V_1.2 currents obtained in the absence of Ca_V_α2δ1. The change in the activation potential could not be solely mediated by a change in the net electrostatic potential. Although Ca_V_1.2 D180A and D180I are likely to alter the net negative charges at the water-accessible interface and to decrease the local Ca^2+^ concentration at the mouth of the channel pore, this effect should result in whole-cell currents being activated at more, not less, negative voltages (36). Furthermore, both Ca_V_1.2 D180E and D181E activated at slightly more positive voltages than Ca_V_1.2 WT despite the conservation of the net negative charge at the site, and this effect was also observed with the double mutant Ca_V_1.2 D180E/D181E (Table 1). These observations demonstrate that physical interaction between the two proteins, as probed by the co-immunoprecipitation studies, is essential for the up-regulation of whole-cell currents. Nonetheless, a negatively aspartate-charged residue is needed at position 180 to promote the Ca_V_α2δ1-induced negative shift in the activation of Ca_V_1.2.

Mutations at adjacent sites confirmed that Asp residues in the IS1S2 play a unique role in the interaction with Ca_V_α2δ1. Whole-cell currents produced by Ca_V_1.2 single mutants at neighboring positions 179 and 182–184 were up-regulated by Ca_V_α2δ1 and activated at potentials generally associated with Ca_V_1.2 WT (Table 1). The results obtained with Ca_V_1.2 Glu-179 mutants (especially E179R) do not support a significant contribution of this residue in establishing an essential interaction with Ca_V_α2δ1.

### Arg-1119 in IIIS5S6 of Ca_V_1.2 contributes modestly to the functional interaction with Ca_V_α2δ1

**Figure 8.**
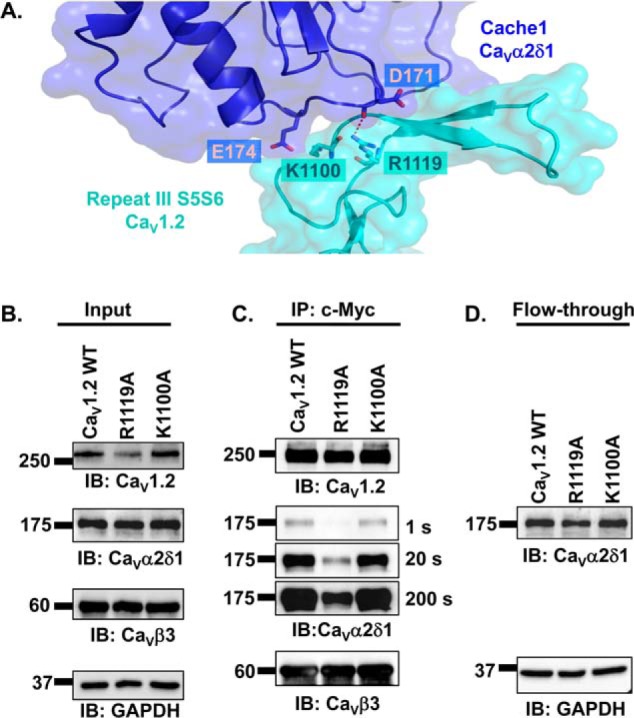
**Extracellular loop S5S6 in repeat III has a modest impact on the interaction between Ca_V_α2δ1 and Ca_V_1. 2/Ca_V_β3 proteins.** *A,* 3D model of IIIS5S6 in Ca_V_1.2 (residues 1059–1204) is shown in *cyan*, and the cache1 domain (residues 104–223) in Ca_V_α2δ1 is shown in *deep blue*. The carboxyl group on the side chain of Arg-1119 in Ca_V_1.2 is located close enough (< 3 Å) to the main-chain atoms of Glu-171 in Ca_V_α2δ1 to potentially contribute to the formation of hydrogen bonds. In contrast, the 3D model does not predict a favorable interaction between Lys-1100 in Ca_V_1.2 and Glu-174 in Ca_V_α2δ1 with a minimum distance estimated to be at 4.6 Å. Modeling was achieved with Modeler 9.17. The figure was produced using PyMOL. *B–D,* HEKT cells were transiently transfected with pmCherry–Ca_V_α2δ1–HA WT and pCMV–Ca_V_β3–c-Myc and either pCMV–Ca_V_1.2 WT, pCMV–Ca_V_1.2 R1119A, or pCMV–Ca_V_1.2 K1100A. Cell lysates were immunoprecipitated overnight with anti-c-Myc magnetic beads to capture Ca_V_β3, eluted in a Laemmli buffer, and fractionated by SDS-PAGE using 8% gels. *B,* immunoblotting (*IB*) was carried out on total proteins (20 μg) collected from the cell lysates for each of the three proteins (Ca_V_1.2, Ca_V_α2δ1, and Ca_V_β3) before the immunoprecipitation assay (*Input*) to confirm that each protein was translated at the expected molecular mass. Each experimental condition is identified by the specific Ca_V_1.2 construct. *C,* immunoblotting was carried out as detailed earlier. Images for Ca_V_α2δ1 were captured after 1, 20, and 200 s of exposure. The signal for the anti-Ca_V_α2δ1 was detected only after a 200-s exposure when probed in the presence of Ca_V_1.2 R1119A/Ca_V_β3. *D,* protein lysates that ran through without binding to the antibody-bead complex (*flow-through fraction*) were collected, diluted in a Laemmli buffer, and fractionated by SDS-PAGE using an 8% gel and revealed with the anti-Ca_V_α2δ1. As seen, mCherry–Ca_V_α2δ1–HA is present in all flow-through fractions at the expected molecular mass (175 kDa) confirming that the proteins were appropriately translated and were present in the preparation in detectable quantities throughout.

In addition to the IS1S2 loop, bioinformatics analysis based upon the 5GJV.PDB structure predicted interaction between Arg-969 and Arg-988 in the IIIS5S6 of Ca_V_1.1 and Asp-173 and Glu-176 in the VWA domain of Ca_V_α2δ1. The reconstructed interface built from the 3D models of IIIS5S6 in Ca_V_1.2 and Ca_V_α2δ1 identifies two positively charged residues in Ca_V_1.2 Arg-1119 and Lys-1100 that could form hydrogen bonds with Ca_V_α2δ1 Asp-171 and Glu-174, respectively (Fig. 8*A*). Within the limits of the 3D model, Ca_V_1.2 Arg-1119 and Ca_V_α2δ1 Asp-171 residues emerge as the most plausible pair to enable protein interaction. Co-immunoprecipitation assays carried out with the single Ca_V_1.2 mutants suggest that Arg-1119 may be involved in the interaction between the two proteins (Fig. 8, *B–D*). Under conditions where Ca_V_1.2 WT, R1119A, and K1100A were well-expressed (Fig. 8*B*), the signal for anti-Ca_V_α2δ1 probed after co-immunoprecipation was slightly reduced for Ca_V_1.2 R1119A.

**Figure 9.**
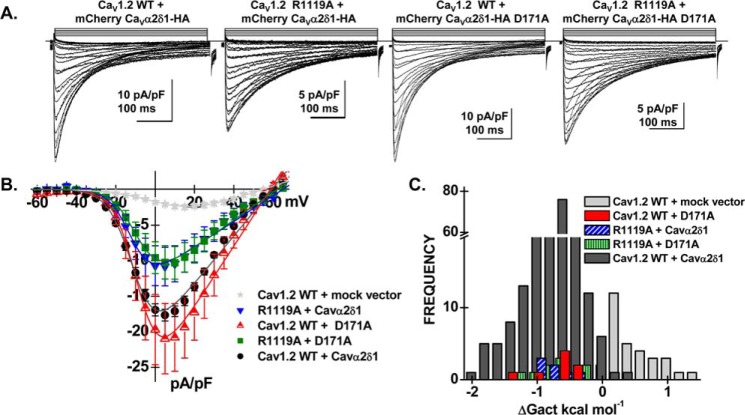
**Double mutant analysis of charged residues in repeat III in Ca_V_1.2.** HEKT cells were transiently transfected with Ca_V_β3, pCMV–Ca_V_1.2 WT or mutant and pmCherry–Ca_V_α2δ1–HA WT or mutant. *A,* representative whole-cell Ca^2+^ current traces were recorded in the presence of 2 mm Ca^2+^ from a holding potential of −100 mV. From *left to right*: Ca_V_1.2 WT with mCherry–Ca_V_α2δ1–HA WT; Ca_V_1.2 R1119A with mCherry–Ca_V_α2δ1–HA WT; Ca_V_1.2 WT with mCherry–Ca_V_α2δ1–HA D171A, and Ca_V_1.2 R1119A with mCherry–Ca_V_α2δ1–HA D171A. Time scale is 100 ms throughout. The current density scale is either 5 or 10 pA/pF as indicated. *B,* averaged current-voltage relationships. Peak current densities *versus* voltage relationships were measured for the WT construct and the mutants (as shown). Current traces obtained with the empty mCherry vector (mock vector) are also shown. Statistical analyses were performed with a one-way ANOVA test: *, *p* < 0.01, and **, *p* < 0.001, against the mock vector. See Table 1 for details. *C,* distribution of the free energies of activation. The values of the free energy of activation (Δ*G*_act_) for all conditions were significantly different from the values measured with the mock vector.

To evaluate whether Ca_V_1.2 Arg-1119 contributes to the functional interaction with Ca_V_α2δ1, a series of patch-clamp experiments were conducted with Ca_V_1.2 R1119A and/or Ca_V_α2δ1 D171A (Fig. 9 and Table 1). As seen, the co-expression of Ca_V_1.2 WT with Ca_V_α2δ1 D171A did not appreciably alter the biophysical properties of Ca_V_1.2 currents. The whole-cell currents obtained in the presence of Ca_V_1.2 R1119A with Ca_V_α2δ1 WT appeared to be roughly 50% lower (*p* < 0.05) than currents obtained with the two WT constructs suggesting that Ca_V_1.2 Arg-1119 contributes to the functional interaction with Ca_V_α2δ1. Co-expressing Ca_V_1.2 R1119A with Ca_V_α2δ1 D171A yielded whole-cell inward currents with biophysical properties similar to Ca_V_1.2 R1119A with Ca_V_α2δ1, suggesting that the interaction may not involve the side chain of Asp-171 in Ca_V_α2δ1. Activation gating was not altered in any of these subunit combinations, and whole-cell currents were seen to activate at the same negative voltage range (Fig. 9*C*). The functional properties of the Ca_V_1.2 K1100A and Ca_V_α2δ1 E174A mutants were also characterized. Results indicate that substituting the charged side chain (in one or both proteins) did not significantly alter the properties of the Ca_V_1.2 whole-cell currents (Table 1). Neighboring mutations at positions 957, 1104, 1109, and 1113 in IIIS5S6 of Ca_V_1.2 were also without effect (Table 1). These results suggest that Arg-1119 in the IIIS5S6 loop of Ca_V_1.2 is the residue the most likely to play a role in the functional interaction with Ca_V_α2δ1, but its role might be more modest than Ca_V_1.2 Asp-181.

## Discussion

### Negatively charged residues in the first extracellular loop control the association with Ca_V_α2δ1

Ca_V_α2δ1 is required to reconstitute the biophysical properties of native L-type channels in cardiomyocytes (22) and is regarded as an intrinsic subunit of Ca_V_1.2 channels (16–20). Ca_V_α2δ1 undergoes a complex series of co- and post-translational modifications (27, 30, 37, 38), and expression at the cell surface of the mature extracellular Ca_V_α2δ1 protein is a prerequisite for formation of the heteromeric complex and channel modulation (30).

Ca_V_α2δ1 produces a 5–10-fold increase in peak current density and promotes channel activation in a physiological range. The agonist-like properties of Ca_V_α2δ1 are manifested by a major impact on channel gating without significant change in the protein density at the cell surface (25, 39) or in the single-channel conductance (40). Ca_V_α2δ1 was shown to double the amount of charges moved during channel activation (41), to increase the channel mean open time in single-channel recordings (40), and to augment the effective charge moved by the voltage sensors from repeats I–III (42).

One of the most perplexing questions entails whether this functional modulation requires direct or allosteric interaction of Ca_V_α2δ1 with the pore or the voltage sensor domains of the Ca_V_α1 subunit (31). Low-resolution cryo-EM structural data of Ca_V_1.2 (43, 44) and more recently higher resolution of the skeletal muscle Ca_V_1.1 channel (20, 28) have revealed that Ca_V_α2δ1 straddles the extracellular loops in repeats I–III of the Ca_V_α1 protein with few interactions predicted between residues in repeat IV of Ca_V_1.2 and Ca_V_α2δ1. Herein, we used a combination of 3D modeling, co-immunoprecipitation data, and electrophysiological recordings to characterize the relative contribution of the extracellular loops in repeats I–III of Ca_V_1.2. Introducing 9-residue epitopes after Ser-182 in IS1S2 prevented the co-immunoprecipitation of the two proteins and the functional modulation of Ca_V_1.2 currents by Ca_V_α2δ1. Our studies reveal that a negatively charged residue is required at position Ca_V_1.2 Asp-181 to mediate physical interaction with Ca_V_α2δ1 and consequently channel modulation. Substitution by glycine, hydrophobic alanine, or positively charged arginine at this position prevented protein co-immunoprecipitation and functional modulation of Ca_V_1.2 currents, whereas the functional properties of Ca_V_1.2 D181E were not significantly different from that observed with the WT construct. These effects were seen to be strongly position-dependent. Whole-cell currents produced by mutating the side chain at position Ca_V_1.2 Asp-180 were up-regulated 3–7-fold by Ca_V_α2δ1. Nonetheless, most Asp-180 mutants activated in the positive range of voltages, typically observed in the absence of Ca_V_α2δ1. Hence, it appears that the nature of the side chain at position 180 in Ca_V_1.2 is critical to convey the Ca_V_α2δ1-mediated shift in the activation potential.

The contribution from extracellular loops connected to the pore region, although not being completely ruled out, can be currently qualified as being modest. Functional modulation and co-immunoprecipitation were not significantly altered when epitopes were inserted in IS5S6 and IIS5S6 of Ca_V_1.2. Furthermore, the substitution of the positively charged Arg-1119 in IIIS5S6 of Ca_V_1.2 decreased the peak current density but did not prevent co-immunoprecipation of the two proteins. These results suggest that there is a smaller but not negligible interaction between IIIS5S6 of Ca_V_1.2 and Ca_V_α2δ1 as proposed previously (42). However, we gathered little evidence for a strong role of the extracellular residues forming part of the external vestibule in the pore of repeat II (IIS5S6).

Our results are qualitatively in agreement with voltage-clamp fluorometry data obtained on Ca_V_1.2/Ca_V_β3/Ca_V_α2δ1 channels showing that the four voltage sensors are not functionally equivalent (45). Experiments from the same group further demonstrated that Ca_V_α2δ1 enhances the charge displacement and promotes the energetic contribution from voltage sensors in repeats I–III (42). Close examination of these data suggest that Ca_V_α2δ1 modifies more significantly the properties of the voltage sensor in repeat II, but these differences may result from changes in the conformations of the specific Ca_V_1.2 and Ca_V_α2δ1 constructs. It is in fact remarkable that the dataset are such in good agreement given the intrinsically distinct experimental designs. The Ca_V_α2δ1 construct we used was tagged with a mCherry fluorophore in its C terminus and a HA epitope in its extracellular cache2 domain, and we have modified key residues in extracellular loops of Ca_V_1.2. Voltage-clamp fluorometry experiments were carried out with the unadulterated Ca_V_α2δ1 protein after the covalent modification of the Ca_V_1.2 protein modified at selected positions in the extracellular loops linking S3 and S4. Future experiments are needed to elucidate further the complex set of interactions and the complete energy landscape at the hydrophilic interface.

### What is the putative mechanism responsible for the Ca_V_α2δ1-mediated modulation of Ca_V_1.2?

Molecular dynamics simulations of our 3D model suggest that Ca_V_1.2 Asp-181 establishes electrostatic interactions with Ser-263 with minor contributions from the adjacent residues Ser-261 and Gly-262 in the VWA domain of Ca_V_α2δ1. Long-range interactions contrast with the nanomolar high-affinity hydrophobic van der Waals interactions at the Ca_V_1.2/Ca_V_β interface, which is anchored by a unique tryptophan residue in the pore-forming subunit (15, 21, 29) facing leucine residues in the guanylate domain of Ca_V_β (46). Polar interactions are often found at the protein interface of loosely connected protein complexes with association constants in the micromolar range (47). Although not identified in the high-resolution cryo-EM structure, it is likely possible that water molecules are present at the interface and play a role in mediating the interaction between the two proteins. The movement of water molecules would in turn increase the number of configurations that the side chains can adopt at the hydrophilic interface.

Within the precision of the cryo-EM structure and the 3D model of the extracellular loops of Ca_V_1.2, we propose that the IS1S2 loop undergoes a conformational change prompted by the binding of Ca_V_α2δ1, which would in turn stabilize salt bridges known to be formed between positively charged S4 residues and negatively charged residues in S2 and S3 transmembrane helices (48, 49). Ca_V_α2δ1 could be required to stabilize the secondary structure of the extracellular IS1S2 loop around Pro-178. In this scheme, negatively charged residues Asp-180 and Asp-181 play essential roles in modifying the energy landscape. First, the interaction of Asp-181 with Ca_V_α2δ1 could stabilize the interaction between residues forming the MIDAS that is required for the function of Ca_V_α2δ1 (50) such that substitution of the negative charge at position 181 in Ca_V_1.2 would destabilize MIDAS. This interpretation is congruent with molecular dynamics simulations indicating that the presence of Ca^2+^ in the MIDAS increases the strength of the non-bonded interactions between residues Ca_V_1.2 Asp-181 and Ca_V_α2δ1 Ser-263. In addition, the strong interactions between Ca_V_α2δ1 and Ca_V_1.2 Asp-181 could promote a long-lived conformation in Ca_V_1.2 that would facilitate the intramolecular interaction of Ca_V_1.2 Asp-180 with the external IS3S4 loop. This interaction could be propagated through water molecules, likely to be abundant at the hydrophilic interface. Alteration in the side chain at Asp-180 could thus compromise its interaction with the IS3S4 loop without perturbing significantly the physical interaction with Ca_V_α2δ1. In turn, the interaction between Asp-180 and the IS3S4 loop could promote a conformation that could lock the S4 voltage sensor into the open state and thus account for the improved activation gating. More experiments are needed to confirm this model and assess its universality in regard to the modulation of high-voltage-activated Ca_V_2 channels by Ca_V_α2δ1. In the context where the molecular properties of the extracellular S3S4 loops are critical determinants of the gating properties in L-type Ca_V_1.1 channels (49), it is also important to note that the atomic coordinates of the IS3S4 loop are missing from the 5GJV.PDB structure and were constructed *de novo* in our virtual model. At this time, we propose that Ca_V_α2δ1 modulates Ca_V_1.2 currents by indirectly exerting its effect on the voltage sensor domain in repeat I rather than on the pore region.

## Experimental procedures

### Recombinant DNA techniques

The rabbit Ca_V_1.2 (GenBank^TM^ X15539) and the rat Ca_V_β3 (GenBank^TM^ M88751) were subcloned in commercial vectors under the control of the CMV promoter as described elsewhere (25, 46). The coding sequence (1091 residues) of the rat brain Ca_V_α2δ1 clone (GenBank^TM^ NM_012919) (51) was subcloned in the pmCherry–N1 vector, and the hemagglutinin (HA) epitope (YPYDVPDYA) was inserted in the extracellular domain of Ca_V_α2 between Asp-676 and Arg-677, such that the HA epitope is accessible from the extracellular medium, and the mCherry is translated after the C terminus. This construct enables the detection of the Ca_V_α2δ1 proteins expressed at the cell surface as described previously (27, 30, 32). Point mutations and insertions were produced with the Q5 site-directed mutagenesis kit (New England Biolabs) in the pCMV–Ca_V_1.2 and in the pmCherry–Ca_V_α2δ1–HA (in Fig. 9) constructs according to the manufacturer's instructions as described elsewhere (25, 27). The HA or the BTX (WRYYESSLEPYPD) epitope tags were inserted between Ser-182 and Asn-183 within the S1–S2 extracytoplasmic loop in repeat I of the Ca_V_α1 subunit of Ca_V_1.2. The HA epitope tag was also inserted in two other sites, between Gln-331 and Glu-332 (S5–S6 loop, repeat I) and between Asp-710 and Glu-711 (S5–S6 loop, repeat II). All HA and BTX constructs were produced with the Q5 site-directed mutagenesis kit (New England Biolabs) according to the manufacturer's instructions. Briefly, large insertions (9–13 amino acids) were performed by incorporating half of the desired insertion into the 5′ ends of both desalted primers. After PCR, a kinase/ligase/DpnI enzyme mix was added to the amplified DNA for circularization and template removal before transformation into high-efficiency DH5-α-competent *Escherichia coli* (New England Biolabs). All constructs were verified by automated double-stranded sequence analysis (Genomics Platform, IRIC, Université de Montréal, Quebec, Canada).

### Immunoblotting of total cell lysates from HEKT cells

Protein expression of all constructs was confirmed by Western blotting in total cell lysates but not shown herein (21, 25, 27). Briefly, HEKT cells were lysed with a RIPA buffer (150 mm NaCl, 1% IGEPAL, 0.5% sodium deoxycholate, 0.1% SDS, 50 mm Tris, pH 8.0) containing a protease inhibitor mixture including 4-(2-aminoethyl)benzenesulfonyl fluoride hydrochloride, aprotinin, bestatin, E-64, leupeptin, and 1 mm EDTA (Sigma) for 30 min at 4 °C. The cell lysates were sonicated and centrifuged at 13,000 rpm for 30 min at 4 °C. Supernatant was collected, and proteins were quantified with the Pierce BCA Protein Assay Kit (Thermo Fisher Scientific, Ottawa, Ontario, Canada). Immunoblotting was carried out with fresh lysates. Proteins were mixed with the Laemmli sample buffer in the presence of 0.4 mm 2-mercaptoethanol and electrophoresed on an 8% SDS-polyacrylamide gel alongside the Precision Plus Protein^TM^ dual-color standard (Bio-Rad). After electroblotting and blocking with 5% (w/v) skim milk for 30 min, the supported nitrocellulose membranes (Bio-Rad) were incubated with the appropriate antibodies. The Ca_V_1.2 mutants were tested with the anti-Ca_V_1.2 (Alomone, Jerusalem, Israel, 1:5000). Membranes were stripped and incubated with an anti-GAPDH as a loading control (Sigma, 1:10,000) unless stated otherwise. Signals were detected with the ECL substrate. Blots were visualized with the ChemiDoc Touch system (Bio-Rad). Molecular weights were estimated using Image Lab^TM^ software version 5.2 (Bio-Rad) by linear regression of standard molecular weight markers.

### Co-immunoprecipitation of Ca_V_1.2 and Ca_V_α2δ1 with Ca_V_β3-c-Myc

HEKT cells were transiently transfected with pCMV–Ca_V_β3-c-Myc and pCMV–Ca_V_1.2 WT or mutants and pmCherry–Ca_V_α2δ1–HA WT. Two days after transfection, cells were homogenized in 20 mm NaMOPS (pH 7.4), 300 mm NaCl, and 1% digitonin supplemented with protease inhibitors (Thermo Fisher Scientific). Homogenates were sonicated, incubated for 1 h at 4 °C, and centrifuged at 16,000 × *g* for 30 min. A fraction (20 μg) of the homogenates or starting material was set aside as the input fraction and was immunoblotted to confirm the presence of all three proteins of interest. Co-immunoprecipitation was carried out using 150 μl of the homogenates containing 3.8 ± 0.1 μg/μl (*n* = 48) of total proteins. This solution (containing 555–585 μg of total proteins) was diluted with an equal volume of 20 mm NaMOPS (pH 7.4), 300 mm NaCl (to 0.5% final concentration of digitonin) and mixed by pipetting. The 300-μl protein solution was incubated overnight with 50 μl of anti-c-Myc magnetic beads (Thermo Fisher Scientific). Beads were collected using a PureProteome magnetic rack (Millipore). The flow-through fraction containing the unbound proteins was conserved, and 15 μl of this protein solution was immunoblotted to validate the presence of Ca_V_α2δ1 in all samples. The magnetic beads were washed three times with a buffer containing 20 mm NaMOPS (pH 7.4), 300 mm NaCl, and 0.2% digitonin. The bound proteins were eluted with 20 μl of Laemmli buffer at 95 °C for 5 min, electrophoresed on an 8% SDS-polyacrylamide gel, and transferred onto a nitrocellulose membrane. Western blotting was carried out with either anti-Ca_V_β3 (Alomone, 1:10,000), anti-Ca_V_1.2 (Alomone, 1:5000), or the anti-Ca_V_α2δ1 (Alomone, 1:1000) with an anti-rabbit as secondary antibody (Jackson ImmunoResearch, 1:10,000).

Signals were detected with the ECL chemiluminescent substrate (Thermo Fisher Scientific), and blots were visualized with the ChemiDoc Touch system (Bio-Rad). Each series of experiments was performed a minimum of three separate times after loading the same quantity of proteins onto the beads.

### Flow cytometry assays

Flow cytometry experiments were carried out to evaluate the cell-surface expression of the mCherry–Ca_V_1.2–HA WT and mutants studied in electrophysiology. Stable Ca_V_β3 cells were transiently transfected with pCMV–Ca_V_α2δ1 WT and pmCherry–Ca_V_1.2–HA WT or mutants where the HA epitope was inserted after Asp-710 (32). Experiments were conducted and analyzed as published before (25, 27, 30) and described in greater details elsewhere (32). Briefly, the cell-surface expression of the mCherry–Ca_V_1.2–HA WT was detected with the FITC-conjugated mouse monoclonal anti-HA epitope tag antibody at 5 μg/ml (Sigma). To determine the total quantity of both intracellular and extracellular expression of the tagged proteins, cells were fixed and permeabilized using BD Cytofix/Cytoperm^TM^ fixation/permeabilization solution kit (BD Biosciences). This procedure was especially important for the mutants that failed to generate significant cell-surface fluorescence as a means to confirm the accessibility of the HA epitope. Roughly 10,000 cells were counted using a FACSAria III® SORP flow cytometer (BD Biosciences). The control conditions were carried out in triplicate with each series of experiments as follows: (*a*) untransfected Ca_V_β3 cells without the anti-HA FITC-conjugated antibody; (*b*) untransfected Ca_V_β3 cells with the anti-HA FITC-conjugated antibody to assess the level of background staining; and (*c*) Ca_V_β3 cells transfected with pCMV–Ca_V_α2δ1 WT and pmCherry–Ca_V_1.2–HA WT, the latter serving as a quality control of transfection. Expressing mCherry–Ca_V_1.2 HA WT in HEKT cells produced a 1-log increase in the FITC (“*x*” axis) and a 3-log increase in mCherry fluorescence (“*y*” axis) on two-dimensional dot plots as shown in Fig. 6*D*.

Although not reported in details herein, flow cytometry data were analyzed using the FlowJo software, version 10 (TreeStar, Ashland, OR) as described (32). Relative expression of the pore-forming subunit of Ca_V_1.2 (Ca_V_α1.2) was calculated based on Δmedian fluorescence intensity (ΔMedFI) for each fluorophore (mCherry or FITC). ΔMedFI for FITC measured in intact non-permeabilized cells was used as a relative index of the steady-state cell-surface expression of the HA-tagged Ca_V_1.2, whereas the ΔMedFI for mCherry attested that the protein was translated until the C terminus. ΔMedFI values were normalized to the maximum value measured the same day for mCherry–Ca_V_1.2–HA WT expressed under the same conditions. The normalized ΔMedFI values for mCherry measured for each mutant in intact and permeabilized cells were not significantly different from one another (*p* > 0.1) (data not shown) suggesting that the cell permeabilization procedure did not distort significantly the relative fluorescence readout under most conditions.

### Patch-clamp experiments in HEKT cells

Whole-cell patch-clamp experiments were carried out on isolated cells after transfection in HEKT cells in the presence of the peGFP vector coding for the green fluorescence protein (GFP) (0.2 μg) as a control for transfection efficiency. Only the GFP-positive cells were patched. Electrodes were filled with a solution containing (in mm) 140 CsCl, 0.6 NaGTP, 3 MgATP, 10 EGTA, 10 HEPES and titrated to pH 7.3 with NaOH with a resistance varying between 2.8 and 3.2 megohms. Cells were bathed in a modified Earle's saline solution containing (in mm) 135 NaCl, 20 TEA-Cl, 2 CaCl_2_, 1 MgCl_2_, 10 HEPES and titrated to pH 7.3 with KOH. GFP-positive cells were selected for patching. On-line data acquisition was achieved with the Axopatch 200-B amplifier (Molecular Devices, Sunnyvale, CA) connected with the PClamp software Clampex 10.5 through the Digidata 1440A acquisition system (Molecular Devices) (25). A series of 450-ms voltage pulses were applied from a holding potential of −100 mV at a frequency of 0.2 Hz, from −60 to +70 mV at 5-mV intervals. Series resistance was compensated to ∼85% after on-line capacitive transient cancellation. Unless stated otherwise, whole-cell currents were sampled at 5 kHz and filtered at 1 kHz. PClamp software Clampfit10.5 was used for data analysis. Mid-potential of activation values (*E*_0.5, act_) was estimated from the peak I–V curves obtained for each channel composition and were reported as the mean of individual measurements ± S.E. (25, 52). The free energy of activation was calculated using the mid-activation potential shown in Equation 1,

(Eq. 1) $$\Delta G_{\text{act}}=z\;\cdot \;F\;\cdot \;E_{0.5,\;\text{act}}$$

where *z* is the effective charge displacement during activation; and *F* is the Faraday constant. The r100 ratio, defined as the ratio of peak whole-cell currents remaining after a depolarizing pulse of 100 ms (*I*_100 ms_/*I*_Peak_), was calculated for each mutant. As there was no significant change in the channel kinetics, these values were not reported herein. To assess for internal consistency, the experiments carried out with novel mutants always included a control experiment performed with pCMV–Ca_V_1.2 WT (pCMV–Ca_V_1.2 WT + pCMV–Ca_V_β3 + pmCherry–Ca_V_α2δ1–HA WT) thus explaining the larger sample size for Ca_V_1.2 WT. Previous experiments confirmed that mCherry–Ca_V_α2δ1–HA WT sustains the functional modulation of Ca_V_1.2 currents (25). Experiments performed under the same conditions yielded peak current densities that could vary by as much as ±35% between each series of transfections. This variation appeared to be essentially linked to minor changes in the cell density at the time of transfection. Data from all experiments performed under the same conditions over a period of 15 months were pooled, and biophysical properties are reported in the Table 1. Experiments were performed at room temperature (22 °C).

### 3D homology modeling

The atomic coordinates of the Ca_V_α1 protein from Ca_V_1.1 (Protein Data Bank 5GJV) were used to explore the 3D structure of Ca_V_1.2. The rabbit Ca_V_1.1 (1873 amino acids) and rabbit Ca_V_1.2 (2171 amino acids) share 69.3% identity (86.3% similarity) in the 1714-residue overlap between 107 and 1806 (Ca_V_1.1 numbering) with major differences found in the N and C termini. Attempts of modeling the four repeats of the Ca_V_α1 protein from Ca_V_1.2 using the cryo-EM coordinates of Ca_V_1.1 failed to yield 3D models with small root mean square deviations due in large part to the gaps in the atomic coordinates of the Ca_V_α1 protein of Ca_V_1.1, namely for amino acids 139–161 in IS3–S4; 343–357 and 372–425 in IS6–IIS1; 669–798 in IIS6–IIIS1; 1076–1103 in IIIS6–IVS1; 1204–1229 in IVS3S4; and 1396–1671 after IVS6. The atomic coordinates of the extracellular loops IS1S4 and IIIS5S6 predicted to interact with Ca_V_α2δ1 were used to build 3D models of these two regions.

The primary sequence between helix S1 and helix S4, in repeat I of the rabbit Ca_V_1.2 (GenBank^TM^ X15539), shares 69.6% identity with the same region of the rabbit Ca_V_1.1. The primary sequence of the VWA domain of the rat Ca_V_α2δ1 (our construct, GenBank^TM^ NM_012919) between amino acids 249 and 439 is perfectly conserved between both species. Amino acids 135–281 in Ca_V_1.2 and amino acids 249–439 of the rat Ca_V_α2δ1 were simultaneously aligned to the atomic coordinates of Protein Data Bank 5GJV using the align2D algorithm in Modeler9.17 (53). Modeler 9.17 was used to generate 100 3D models, and the models with the lowest values for the molpdf parameters and discrete optimized protein energy (DOPE) score (54), as reported in the log file, were selected. The DOPE parameter is a statistical potential used to access the energy of the protein model generated through many iterations by Modeler, which produces homology models by the satisfaction of spatial restraints. The gaps of the cryo-EM structure in the extracellular loop linking helix S3 and helix S4 were filled in using PHENIX (phenix.refine) (55) and further refined using the “automatic loop refinement” tool in Modeler. Modeler was used to generate 100 3D models, and the models with the lowest values for the molpdf parameters and DOPE score (54) were selected for this study.

The 3D model of the extracellular loop between transmembrane segments 5 and 6 in repeat III (IIIS5S6) in Ca_V_1.2 was built using Modeler9.17 (53) using a similar strategy. This 145-residue gapless stretch in Ca_V_1.2 (residues 1059–1204) shares 74% identity with Ca_V_1.1. The best model with the lowest value of DOPE score and an average root mean square deviation of 0.4 Å was selected. The protein/protein interfaces were reconstructed in PyMOL. The 3D model of IIIS5S6 in Ca_V_1.2 and the 3D model of the cache1 domain (104–233) of the rat Ca_V_α2δ1 were superimposed with the cryo-EM structure of the Ca_V_1.1 protein complex (PDB code 5GJV). The reconstructed interface shows that residues Lys-1100 and Arg-1119 could form hydrogen bonds with Glu-174 and Asp-171, respectively, in the VWA domain of Ca_V_α2δ1.

Molecular dynamics simulations were performed using CHARMM-CGENFF with explicit solvent water molecules. The system comprises the external loops IS1S2 and IS3S4 of Ca_V_1.2 extending from Ala-172 to Glu-190 and Glu-243 to Val-263, respectively, plus the VWA domain of the rat Ca_V_α2δ1 extending from Ala-249 to Ala-441. Identification of the water-exposed external loops was based on the PPM server calculations, which determine the rotational and translational positions of transmembrane and peripheral proteins in membranes using their 3D structure (PDB coordinate file) as input as seen in http://opm.phar.umich.edu/server.php^4^ (57). The system was solvated in a 84.5 Å side cubic cell containing 19 938 TIP3P model water molecules. Altogether the system consisted of 63,938 atoms, including 60 K^+^ and 56 Cl^−^ ions to ensure electroneutrality at near physiological concentrations. Cut-on and cut-off parameters needed to define non-bonded interactions were set to 10 and 12 Å, respectively, and SHAKE constraints were used to determine the lengths of bonds involving hydrogen atoms. Constraints with a force of 10 were applied to the end residues of IS1S2 of Ca_V_1.2 (Ala-172 and Glu-190), IS3S4 (Glu-243 and Val-263), and the VWA domain (Ala-249 and Ala-441) to account for the anchoring of the IS1S2 and IS3S4 loops to their respective transmembrane segments and the folding of the VWA domain between the cache1 and the cache2 domains. Trajectories were generated for 20 and 25 ns (in the presence and in the absence of Ca^2+^, respectively) using a time step of 2 fs, and electrostatic and van der Waals interaction energies computed from trajectories were sampled at 0.01 ns. Molecular dynamics simulations were performed for a system at constant pressure (1 atm) and constant temperature (303 K).

### Statistics

Results were expressed as mean ± S.E. Tests of significance were carried out using the unpaired ANOVA with the Tukey Test embedded in the Origin 7.0 analysis software (OriginLab Corp., Northampton, MA). Data were considered statistically significant at *, *p* < 0.01, and **, *p* < 0.001.

## Author contributions

B. B. conducted and analyzed the patch-clamp experiments. J. B. was responsible for the homology modeling and carried out the co-immunoprecipitation assays. M. P. T. performed and analyzed flow-cytometry experiments. B. B. and M. P. T. produced the mutants. B. B., J. B., and M. P. T. performed standard immunoblotting assays. R. S. supervised the homology modeling and performed molecular dynamics simulations. L. P. coordinated the study, interpreted the data, and wrote the manuscript. All authors contributed to the design of the experiments, reviewed the results, and approved the final version of this manuscript.

## Footnotes

4 Please note that the JBC is not responsible for the long-term archiving and maintenance of this site or any other third party hosted site.

## Footnotes

3 The abbreviations used are:

VWA: von Willebrand factor A
AID: α-interacting domain
BTX: bungarotoxin
Δ*G*_act_: free energy of activation
HEKT: human embryonic kidney 293 cells stably expressing an SV40 temperature-sensitive T antigen
MIDAS: metal-ion-dependent adhesion site
ΔMedFI: relative median fluorescent intensity
ANOVA: analysis of variance
pF: picofarad
DOPE: discrete optimized protein energy
PDB: Protein Data Bank.
